# *Drosophila* Adaptation to Viral Infection through Defensive Symbiont Evolution

**DOI:** 10.1371/journal.pgen.1006297

**Published:** 2016-09-29

**Authors:** Vitor G. Faria, Nelson E. Martins, Sara Magalhães, Tânia F. Paulo, Viola Nolte, Christian Schlötterer, Élio Sucena, Luis Teixeira

**Affiliations:** 1 Instituto Gulbenkian de Ciência, Oeiras, Portugal; 2 Centre for Ecology, Evolution and Environmental Changes (cE3c), Faculdade de Ciências, Universidade de Lisboa, Lisboa, Portugal; 3 Institut für Populationsgenetik, Vetmeduni Vienna, Wien, Austria; 4 Departamento de Biologia Animal, Faculdade de Ciências, Universidade de Lisboa, Lisboa, Portugal; Fred Hutchinson Cancer Research Center, UNITED STATES

## Abstract

Microbial symbionts can modulate host interactions with biotic and abiotic factors. Such interactions may affect the evolutionary trajectories of both host and symbiont. *Wolbachia* protects *Drosophila melanogaster* against several viral infections and the strength of the protection varies between variants of this endosymbiont. Since *Wolbachia* is maternally transmitted, its fitness depends on the fitness of its host. Therefore, *Wolbachia* populations may be under selection when *Drosophila* is subjected to viral infection. Here we show that in *D*. *melanogaster* populations selected for increased survival upon infection with *Drosophila* C virus there is a strong selection coefficient for specific *Wolbachia* variants, leading to their fixation. Flies carrying these selected *Wolbachia* variants have higher survival and fertility upon viral infection when compared to flies with the other variants. These findings demonstrate how the interaction of a host with pathogens shapes the genetic composition of symbiont populations. Furthermore, host adaptation can result from the evolution of its symbionts, with host and symbiont functioning as a single evolutionary unit.

## Author Summary

Animals live in close association with microbial partners that can shape many aspects of their lives. For instance, several insects carry bacteria that defend them against parasites and infectious diseases. The intracellular bacterium *Wolbachia* protects the fruit fly *Drosophila melanogaster* against viral infection. Natural populations of *Drosophila* carry different variants of *Wolbachia*, which differ from one another in the strength of this protection. Here we show that a population of *Drosophila* infected with viruses during several generations adapts to this challenge through turnover in *Wolbachia* composition. The *Wolbachia* variants that give higher protection to viruses, by increasing fly survival and fecundity upon infection, are strongly selected. This work demonstrates that the interaction of an animal with a pathogen can shape its associated microbial populations. We show that adaptation to pathogens can be achieved not only through selection of resistance on the host proper but also through the evolutionary shaping of its microbial community.

## Introduction

Animals and plants live in close association with numerous symbiotic bacteria that often cause strong phenotypic changes in their hosts [1]. For example, defensive symbionts can increase host resistance to pathogens and parasitoids [2–8]. In insects, several defensive symbionts are maternally transmitted [3–7], such that the fitness of the symbiotic bacteria is dependent on that of their female hosts. Therefore, one can expect that selection on host phenotypes, including resistance to (other) parasites, impacts the evolution of the bacterial symbiont population.

Host parasite burden can impact symbiont populations. For example, experimental evolution of the pea aphid *Acyrthosiphon pisum* or of *Drosophila hydei* in the presence of parasitoid wasps, caused an increase in the frequency of individuals carrying the protective symbionts *Hamiltonella defensa* and *Spiroplasma*, respectively [9,10]. Also, the recent spread of a *Spiroplasma* symbiont in natural populations of *D*. *neotestacea* in North America has been associated with the arrival of a parasitic nematode to this continent [7]. In agreement with this, the frequency of *Spiroplasma* in a *D*. *neotestacea* population increases in the presence of the parasitic nematode during experimental evolution [11]. These studies show changes in the prevalence of endosymbiont infection in host populations, but do not address selection at the level of the genetic diversity of the symbiont itself. However, some evidence suggests that this could be the case: 1) some defensive symbiont populations display genetic and phenotypic variability [12–18] and 2) variants or strains of endosymbionts change in frequency in natural populations or during experimental evolution [19–21]. Nonetheless, a clear link between the selective pressure exerted on hosts and the genetic changes observed in the symbionts has been missing. In this study, we establish a relation between host adaptation to parasites and changes in the genetic composition of endosymbiont populations.

*Wolbachia* is a maternally-transmitted bacterial endosymbiont widespread in arthropods [22]. In some natural hosts it induces strong protection against infection with several RNA viruses [3,4,23,24]. Importantly, genetic variation in the *Wolbachia* strain of *Drosophila melanogaster* (*w*Mel), can be linked to the strength of antiviral protection [14,15]. Using experimental evolution, we have previously shown that *D*. *melanogaster* populations adapt to *Drosophila* C virus (DCV) challenge [25]. Resistance to this pathogen increases over twenty generations and we identified the genetic bases of this adaptation at the host level [25]. However, all individuals of the outbred founder population carried *Wolbachia* [26]. Therefore, we used this unique setup to ask if the genetic composition of the *Wolbachia w*Mel populations also changed during host adaptation to DCV challenge and whether this change could impact on *Drosophila* fitness.

## Results

**Fig 1:**
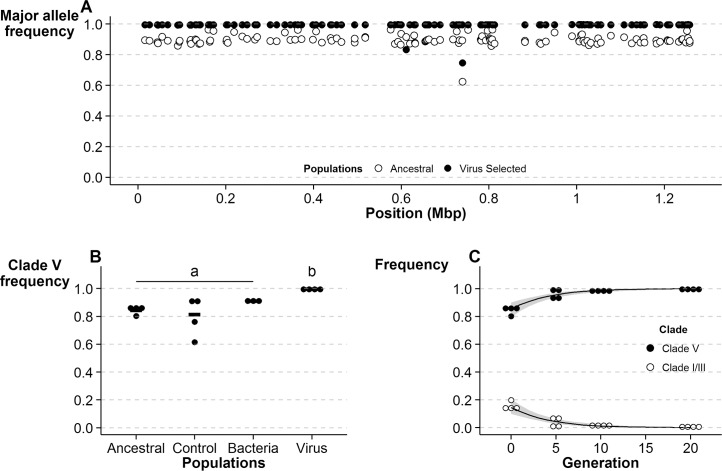
Selection of *Wolbachia w*Mel Clade V variants after experimental evolution of *Drosophila melanogaster* with DCV. **(A)** Frequencies of the major allele of *Wolbachia* single nucleotide polymorphisms (SNPs) in Ancestral and Virus-Selected populations, determined by Pool-Seq. All SNPs with significantly different frequencies at generation 20 between Ancestral (open circles) and Virus-Selected populations (closed circles) are shown. **(B)** Frequencies of flies carrying Clade V *w*Mel variants in Ancestral, Control, Bacteria-Selected, and Virus-Selected populations (last three at generation 20). 96 individual flies from each population were tested for a clade V diagnostic SNP at position 805,011. Each data point represents the proportion of flies carrying clade V *w*Mel in a population. Letters (a,b) refer to statistically homogenous groups of mean Clade V frequencies, based on Tukey’s pairwise comparisons between all populations (*p* > 0.23 within all group “a” populations, *p* < 0.003 for all comparisons with Virus-Selected populations). **(C)** Frequency of flies carrying Clade V (closed circles) or Clade I/III (open circles) variants in Ancestral (generation 0) and Virus-Selected populations at generations 5, 10 and 20. These frequencies were determined from 96 individuals from each replicate population, as in (B). Black solid line and gray shading represents the best fit for the logistic regression and 95% Confidence interval (CI), respectively.

We performed experimental evolution on four replicate populations of *D*. *melanogaster* under selection with systemic DCV infection (Virus-Selected) and four replicates with mock infection (Control) [26]. DCV infection was performed at every generation using the same virus strain, at the same dose. As previously described [25], we performed genome-wide sequencing of DNA from pools of each population (Pool-Seq) [27,28]. Using Pool-Seq on the Ancestral populations and on the Control and Virus-Selected populations after 20 generations [25], we determined the genetic diversity of *Wolbachia* in these populations. We found statistically significant changes in the frequency of 125 single nucleotide polymorphisms (SNPs) between the Ancestral and the Virus-Selected populations (Fig 1A, S1 and S2 Figs, S1 Dataset). Of these, 111 were also significantly different between Control and Virus-Selected populations, but not between Control and Ancestral populations, showing that these changes in the genetic composition of the *Wolbachia* populations are mostly specific to the response to viral infection.

Phylogenetic analysis, based on whole genome sequencing of *Wolbachia* and mitochondria, indicate that in the recent past *w*Mel has been strictly vertically transmitted [13,29,30]. Moreover, there is no evidence of fly lines simultaneously carrying *w*Mel variants from distant haplotypes or recombination between these [29]. Therefore we inferred *Wolbachia* haplotypes in the Ancestral, Control and Virus-Selected populations from the Pool-Seq data (S1 Text). Overall, we identified diagnostic SNPs (i.e. SNPs present in all variants, and only in variants, of a specific clade) for three of the major clades of *w*Mel (S1 Dataset) [13,14,21]. The Ancestral *Wolbachia* populations consisted of approximately 88% clade V variants and 12% of variants of clades I and III. In the Virus-Selected populations all these diagnostic SNPs became fixed with the nucleotide that matches clade V (S1 Dataset). In fact, in all the 123 SNPs that became fixed between Ancestral and Virus-Selected populations the fixed nucleotides match clade V. Moreover, between Ancestral and Control populations, the 8 SNPs that significantly changed have only been detected before in clades I and III variants, whereas Clade V specific SNPs did not significantly change in frequency between these populations (S1 Text and S1 Dataset). Therefore, we conclude that selection of *D*. *melanogaster* with a viral challenge changed the frequencies of *w*Mel variants in the host populations and led to fixation of clade V *w*Mel variants.

To confirm that the fixation of clade V variants was specific to the Virus-Selected populations, we analyzed individual flies from the Ancestral, Control and Virus-Selected populations as well as from a parallel selection regime in which *Drosophila* was challenged with systemic bacterial infection [26] (Fig 1B). We determined the *w*Mel variant carried by 96 individual flies from each replicate population through restriction analysis of a PCR fragment containing a clade V diagnostic SNP. This analysis distinguishes flies carrying *w*Mel variants of clades I/III or clade V. The frequencies of flies carrying clade V *w*Mel variants in Ancestral, Control and Virus-Selected populations are in agreement with the Pool-Seq data (S1 Text) and clade V variants are only fixed in the Virus-Selected populations. We observed significant differences in frequencies between the Virus-Selected populations and the other tested populations but not between any other regimes (generalized linear mixed model (GLMM), Selection Regime effect, χ^2^_3_ = 31.648, *p* < 0.001, Tukey HSD, |z| > 3.437, *p* < 0.005 for all comparisons with the Virus-Selected populations, |z| < 2.067, *p* > 0.23 for all other comparisons). These data argue against drift being responsible for the fixation of clade V variants since Bacteria-Selected populations had fewer surviving individuals for a larger number of generations than Virus-Selected populations (S1 Text and S2 Dataset) and *w*Mel variants of clade V did not reach fixation in any of the Bacteria-Selected populations. Moreover, a time-course analysis of *w*Mel clade V frequencies in the Virus-Selected regime, based on individual genotyping, also shows that changes in frequencies were parallel in all four replicates (Fig 1C). Finally, based on the frequencies of clade I/III and clade V variants in generations 0, 5, 10 and 20 we estimated a strong selection coefficient against clade I/III variants of 0.263 (0.177–0.349) (estimated log-linear slope using GLMM, Generation effect, χ^2^_1_ = 42.466, *p* < 0.001) [31]. Therefore, fixation of clade V variants in all the Virus-Selected populations is unlikely due to drift, injury or a generic immune challenge, but the consequence of the specific adaptation to viral challenge.

**Fig 2:**
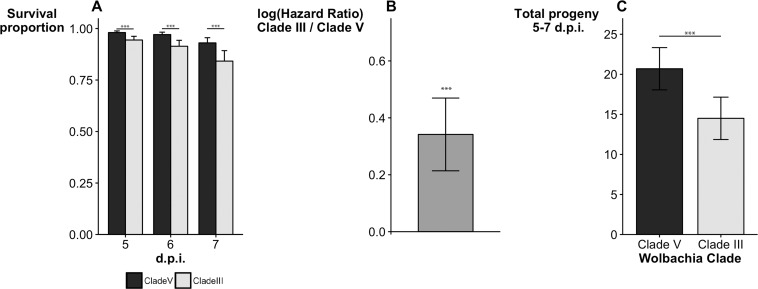
***Wolbachia w*Mel Clade V variants confer higher protection to viral infection when compared with clade III variants (A)** Survival of flies carrying clade V and clade III *w*Mel variants five, six and seven days post infection with DCV (d.p.i.). **(B)** Cox hazard ratio of flies carrying clade III *w*Mel variants compared with flies carrying clade V, calculated from survival data until 20 d.p.i. **(C)** Reproductive output of parents 5–7 d.p.i. In all assays the female progeny of eleven independent reciprocal crosses between isofemale flies, carrying Clade V and Clade III *w*Mel variants, were analyzed after systemic infection with DCV (2 x 10^7^ TCID_50_/ml). ***-*p*< 0.001. Means (± 95% confidence intervals) are shown in all panels.

To analyze the phenotype of clade V *w*Mel variants against clade III variants we established eleven different isofemale lines carrying *w*Mel from clade V and eleven different isofemale lines carrying *w*Mel from clade III. These lines were established from the Control populations. To directly compare the differences between *w*Mel from the different clades we set up reciprocal crosses between eleven independent pairs of clade III and clade V isofemale lines. Since *w*Mel is only maternally transmitted, the female progeny of each of these paired crosses differed in the *w*Mel variant, but had the same host genotype. During the virus-selection protocol, reproduction of surviving adults took place five to seven days after DCV infection [25]. At five, six and seven days after DCV infection, flies carrying *w*Mel clade III variants had lower survival than flies with clade V variants (Fig 2A, GLMM, *w*Mel clade effect, χ^2^_1_ > 16.44, *p* < 0.001 in all daily comparisons, see also analysis of S3A Fig, below). Analysis of the survival data until 20 days post-infection confirms an overall lower susceptibility upon viral infection of flies with *w*Mel variants of clade V compared with flies carrying clade III variants (Fig 2B, mixed effect Cox model, *w*Mel clade effect, χ^2^_1_ = 25.817, *p* < 0.001, see also analysis of S3B Fig, below).

We also analyzed the reproductive output of flies with the different *w*Mel variants (from the same reciprocal crosses), five to seven days post-infection with DCV (Fig 2C). Flies with clade III variants had fewer progeny than flies carrying clade V variants (linear mixed model (LMM), *w*Mel clade effect, χ^2^_1_ = 39.217, *p* < 0.001). This difference between variants is contingent on viral infection, since their reproductive output is not significantly different in the absence of infection (LMM, χ^2^_1_ = 2.321, *p* = 0.128, S4 Fig). The differences between flies carrying *w*Mel clade III variants and flies carrying clade V based on reproductive output and survival at five to seven days post-infection could explain the relative fitness of 0.723 (0.651–0.823) calculated from the above estimated selection coefficient (*w* = 1-*s*).

Mitochondria are co-inherited with *Wolbachia*. Therefore, the phenotypic differences we observed between flies carrying different *w*Mel variants could, hypothetically, be due to phenotypic differences of their associated mitochondria variants. If this were the case, selection could have acted on the mitochondria and indirectly affected frequencies of *Wolbachia* variants. To test for the contribution of mitochondria to the phenotypic differences observed, we repeated these assays with the same isofemale lines and matching isofemale lines from which *w*Mel was removed by tetracycline treatment. We found a significant interaction between *w*Mel/mitochondria clade (cytotype) and *Wolbachia* presence, both in survival 5, 6 or 7 days after infection and in overall survival (S3A and S3B Fig–clade by presence of *Wolbachia*, GLMM and Mixed effect Cox model, *p* < 0.001). Importantly, both models showed a significant difference in survival between flies carrying *w*Mel clade V or *w*Mel clade III, but not between flies of the same cytotypes without *Wolbachia* (pairwise comparisons between clades in Mixed effect Cox Model, |z| = 5.739, *p* < 0.001 and |z| = 0.868, *p* = 0.385, for flies with and without *Wolbachia*, respectively and in pairwise comparisons between clades using GLMM at 5, 6 or 7 days post-infection |z| > 3.794, *p* < 0.001 and |z| < 1.678, *p* > 0.093, for flies with and without *Wolbachia*, respectively). Analysis of differential reproductive output had similar results. There was a significant interaction between cytotype and *Wolbachia* presence (S3C Fig, LMM, clade by presence of *Wolbachia*, χ^2^_1_ = 4.2, *p* = 0.040). Pairwise comparisons of reproductive output between cytotypes with *Wolbachi*a showed a significant difference (*t* = 4.27, *p* < 0.001) but not between cytotypes in the absence of *Wolbachia* (*t* = 1.2, *p* = 0.087). Overall, these data indicate that there is no significant difference in survival or reproductive output, upon viral infection, between flies only carrying different mitochondria. Therefore, the phenotypic differences we observe are due to differences between *w*Mel variants and not between mitochondria variants.

Finally, we tested if lower fitness upon viral infection of flies carrying *w*Mel clade III variants was associated with higher DCV load as different *w*Mel variants have been shown to confer differential resistance to DCV infection [14] (S5A Fig). Flies carrying these variants had 5.4 fold higher levels of DCV compared with flies carrying clade V variants (log-LMM, *w*Mel variant effect, χ^2^_1_ = 11.479, *p* < 0.001). The lower resistance to viruses of flies carrying Clade III variants, compared to Clade V, may explain their lower survival and fertility upon infection. Flies with Clade III variants also had lower *Wolbachia* levels when compared with flies carrying clade V variants (S5B Fig, LMM, χ^2^_1_ = 16.292, *p* < 0.001). This may explain lower antiviral resistance of these variants, in line with previous findings [14,15,32,33].

## Discussion

Our data show that (a) the frequencies of *Wolbachia* variants specifically change when *Drosophila* populations evolve in the presence of viruses, (b) this exposure to DCV leads to fixation of clade V *w*Mel variants, and (c) genetically identical individuals are more protected against DCV infection and display lower viral loads when they harbor these Clade V variants, relative to when they harbor other variants still present in the Control (and Ancestral) population. Moreover, the selection coefficient inferred from the evolutionary dynamics of clade V in DCV-exposed populations could be explained by the fitness advantage of clade V over clade III *w*Mel variants in flies subjected to DCV infection. These results demonstrate that host infection by parasites can be a selective force leading to genetic changes in the endosymbiont population such that the most protective variants become fixed. In turn, this evolution can contribute to host adaptation to pathogens.

We have previously identified two regions in the *D*. *melanogaster* genome that mediate adaptation of this population to DCV infection [25]. Here we show that this adaptation also leads to change in *w*Mel genetic diversity. There may be interactions between selection on the genomes of the symbiont and the host, which we did not test here. We have demonstrated before that the Virus-Selected population had a higher survival upon DCV infection than the Control populations even when *Wolbachia* was removed from these populations [25]. This indicates that, overall, the selected alleles confer an advantage in the presence of viruses independently of the presence of *Wolbachia*. However, it was recently shown that the strength of selection on host genetic variation is decreased in the presence of these protective symbionts [34]. Therefore, the presence or absence of *Wolbachia* interacts with selection at the host level. However, this does not address interactions between selection acting both at the level of the symbiont and the host. We show differences between the *w*Mel variants using isofemale lines established from the Control populations, and therefore not evolved under Virus challenge. This indicates that the virus susceptibility phenotypes associated with the *w*Mel variants are not dependent of selection at the level of the host genome. Moreover, we compared the phenotypes of the progeny of several independent reciprocal crosses between lines carrying different *w*Mel variants. This setup controls for differences in host genetic background. It will be interesting in the future to investigate how genetic variation in the host impacts on the phenotypes of *Wolbachia* variants, and vice-versa.

Other *w*Mel variants were shown to differ in survival upon viral infection [14]. *w*Mel variants from clade VI confer more protection to viruses than variants from clade III or clade VIII [14]. Here clade V variants are more protective than clade III variants (and clade I variants are also counter-selected in the Virus-Selected populations). These results indicate that clade V and VI are more protective against viral infections and clade I, III and VIII, less protective. It will be important in the future to make a direct comparison of the antiviral protection conferred by these different variants and understand their dynamics in natural populations.

Previous work showed that variants that differ in protection to viruses also differed in the cost to the host in the absence of infection, indicating a trade-off between the two traits [14,15]. This led to the suggestion that the frequencies of different variants in natural populations might depend on the prevalence of viruses [14]. Here we demonstrate that an increase in viral burden does lead to changes in *w*Mel variant frequencies. Moreover, the selection coefficient for specific *w*Mel variants can be very high and promote their rapid fixation. *w*Mel variants are strictly maternally transmitted and show no sign of recombination [13,29,30]. Therefore, as in these conditions specific haplotypes are fixed, the overall genetic diversity of *w*Mel is strongly reduced (since mitochondria are co-inherited with *Wolbachia* this selection may also impact on their genetic diversity).

Viruses seem to impose strong natural selective pressure, as demonstrated by the fast evolutionary rates and signatures of positive selection in *D*. *melanogaster* genes involved in antiviral resistance [35]. *Wolbachia* can protect hosts against several positive sense single-stranded RNA viruses [3,4,23,24], including DCV, a natural pathogen of *D*. *melanogaster* [36–39]. However, approximately 25 different viruses have been found to infect natural populations of *D*. *melanogaster* [38,40,41]. Although most of them are positive sense single-stranded RNA viruses we do not know which represent the biggest burden to natural populations. Moreover, the effect of *Wolbachia* against most of these viruses is unknown, although it protects against the few that were tested (DCV, Cricket Paralysis virus, and Nora virus [3,4]). Different *w*Mel variants also have different costs in the absence of infection and this is most probably an important factor in the dynamics of *w*Mel in natural populations [14]. Our particular experimental evolution setup, with all the individuals being infected with DCV at every generation before reproduction, demonstrates that *w*Mel selection upon viral infection is possible. In which conditions and to which degree this occurs in natural populations remains to be determined.

We can explain the strong selection coefficient for clade V over clade III *w*Mel variants with the differences in the protection to viruses they confer to their hosts. Previous analyses of virus-infected hosts carrying different *w*Mel variants or *Wolbachia* strains have shown differences in viral titers and survival [14,15,23,32,33,42]. Here we also show that flies carrying clade V variants have lower viral titers and higher survival when compared to flies carrying clade III variants. This higher survival most likely contributes to the selection of clade V variants. However, there is also a much higher fertility of flies carrying clade V *w*Mel variants, upon viral infection, which likely also determines the strong selection coefficient. In fact, in natural populations this parameter might be more important for the protective effect of *Wolbachia* against viruses and the differential selection of *w*Mel variants, than the effect on host survival.

Here, using experimental evolution, we provide direct proof that endosymbiont and host can form an evolutionary unit with adaptation relying on the evolution of both genomes. It is straightforward to extrapolate our results with maternally transmitted *Wolbachia* to interactions involving other defensive endosymbionts such as *Spiroplasma*, *Regiella*, and *Hamiltonella* [6,7,16]. The tight association between endosymbionts and their hosts make it probable that it is common for selection at the host phenotypic level to impact symbiont population genetics. It will be interesting in the future to assess to which degree this phenomenon occurs in interactions between hosts and microbes with different modes of transmission. One obvious example is the gut microbiota of mammals, which can protect the host against gut pathogens [8] and show some degree of vertical transmission [43]. As research on microbiota-induced phenotypes and potential co-evolution with hosts increases, a central question arises on how selection on the microbiota-induced phenotypes impacts the population genetics of the microbes.

## Materials and Methods

### Foundation, maintenance, and selection of populations

We used an outbred population of *D*. *melanogaster* established in 2007 from 160 fertilized females, as described in [25,26]. The population was kept in laboratory conditions for more than 50 non-overlapping generations at high census. Before the initiation of experimental evolution, this population was serially expanded for two generations to allow the establishment of 36 new populations of which 12 were used in this work. All individual founders were naturally infected with *Wolbachia w*Mel and the initial populations were 100% infected with *Wolbachia* (checked individually by PCR with *wsp* primers, as described in [44]).

Flies were kept in laboratory cages at constant temperature (25°C) and humidity (70%) in a light-darkness cycle (12h:12h). Flies were raised in standard cornmeal-agar medium. Each generation took three weeks and egg density per food cup was controlled.

Virus-Selected populations were infected every generation by pricking flies in the thorax with DCV (2 x 10^7^ median tissue culture infective dose (TCID_50_) per ml)) [25]. DCV was grown and titrated as described in [3]. This dose caused in the initial population an average mortality of 66% 10 days after infection. Three hundred and ten males and 310 females were infected with DCV at every generation. Surviving individuals mated randomly in population cages and eggs were collected five to seven days post-infection. This selection protocol proceeded for 20 generations before Pool-Seq analysis.

Control populations were pricked at every generation with sterile solution. These populations were controlled to 600 adults at every generation.

Bacteria-Selected populations infection and selection protocol at every generation was the same as for the Virus-Selected populations. Flies were infected by pricking with *Pseudomonas entomophila* at a dose that causes an average mortality of 66% in the initial populations (OD600 = 0.01) [26].

### Whole-genome sequencing of populations (Pool-Seq)

DNA extraction, library preparation and whole genome sequencing of pools of individuals was described in [25]. Briefly, 12 populations were sequenced (four per regime): Ancestral (generation 0), Control and Virus-Selected populations, the latter two at generation 20. Genomic DNA was extracted from a homogenate pool of 200 individuals of each population using a high-salt extraction protocol. Genomic DNA was sheared using a Covaris S2 device (Covaris, Inc.) and paired-end 100bp libraries were prepared using the TruSeq v2 DNA Sample Prep Kit (Illumina). Libraries were sequenced on a HiSeq 2000 (Illumina).

Raw reads were trimmed using Trimmomatic [45] (leading and trailing bases clipped if quality < 20, 3’ clipped if average quality of a window (4 bp) dropped below 20, minimum read length = 50) and then realigned to the reference *Wolbachia* genome (NC_002978.6 [46]) using bwa 0.6.2 [47], with the following parameters: maximum differences = 1%, maximum number of gaps = 2, maximum gap or deletion size = 12, seeding disabled. Alignments were converted to the sam/bam format using samtools [48] and sorted, filtered for quality, proper pairs and duplicate reads using bamtools [49]. Afterwards, SNPs were called simultaneously in all populations using freebayes (v 9.9.2) [50], in positions with a minimum count of the alternate allele of 2 and a minimum global alternate allele frequency of 2%. Only biallelic SNPs were considered.

Effects of the polymorphisms on putative coding sequences were predicted using SnpEff 4.11 [51], based on the ENSEMBL GCA_000008025.1.26 genome annotation.

### Determination of frequencies of clade V *w*Mel variants

We analyzed the frequency of clade V *w*Mel variants by testing individual flies in Ancestral, Virus-Selected (at generations 5, 10, and 20), Control (at generation 20), and Bacteria-Selected (evolved against *Pseudomonas entomophila*, at generation 20) populations.

We extracted DNA from 96 individual female flies of each replicate population following the protocol in (http://www.drosdel.org.uk/molecular_methods.php#prep) [52]. Briefly, single flies were squashed in 100 mM Tris-EDTA-NaCl buffer (pH 7.7), 0.5% SDS and incubated at 65°C for 30 minutes. After protein and RNA precipitation with 6M LiCl / 5M KAc, DNA was precipitated using ice-cold isopropanol followed by ethanol cleaning. PCR amplification of the genomic region surrounding position 805,011 was performed using the primers 805011F (5’-AGTCGGGAGCATGAGGGAAAAGT-3’) and 805011R (5’-TTTCAGCATCAGTCGCCTCCGC-3’). The polymorphism was detected by differential cleavage of amplified product with the enzyme *Bts*CI (NEB). Digestion was performed at 50°C for 60 minutes and the digestion product visualized in an agarose gel. The polymorphism at this position distinguishes *w*Mel variants of clades I, II, III and IV from variants of clades V and VI. In our populations this SNP allows distinguishing clade V variants from clade I/III variants.

### Establishment of isofemale lines carrying *w*Mel of clades III and V

Ninety-six isofemale lines were founded from Control populations. The Pool-Seq data show that these populations only had *w*Mel variants from clades III and V.

Each line was tested for three different *w*Mel SNPs. Position 805,011 was tested as above. The SNPs at positions 655,839 and 1,027,577 distinguish clades I, II and III from clades IV, V, VI and VIII. PCR amplification of the genomic regions surrounding these positions were performed using the primers 655839F (5’-AGCAGCTCTAGCAATCGCAGCA-3’), 655839R (5’-GGCGTTTTAGGGGTGTGGTTGGT-3’), 1027577F (5’-TCCTGCATCAGTCCTGCCACCA-3’), and 1027577R (5’-GGCAGCACTGTAGGCTTGACCA-3’). The PCR products were digested at 37°C for 60 minutes using the restriction enzymes *Msc*I and *Hin*dIII (NEB) for positions 655,839 and 1,027,577, respectively. The results of the three enzymes were congruent allowing us to identify isofemale lines carrying clade V or clade III *w*Mel variants.

We also tested for the insertion IS5-WD1310 by PCR, as described in [12]. This insertion is present in clade VI variants, absent in clade III and VIII variants, but unknown for variants of other clades, including clade V [12,14]. All flies were negative for this insertion.

After these analyses we selected eleven independent isofemale lines carrying clade V *w*Mel variants and eleven independent isofemale lines carrying clade III *w*Mel variants. Isofemale lines were kept in vials in similar conditions to the *D*. *melanogaster* populations.

### Generation of flies carrying different *w*Mel variants for phenotypic characterization

Eleven independent pairs of isofemale lines with *w*Mel variants of clades III and V were crossed in a reciprocal scheme (female clade V x male clade III and female clade III x male clade V). The female progeny of these two crosses have an equivalent genetic background but different *w*Mel variants (which is maternally transmitted). This female progeny was used for the phenotypic characterization and each reciprocal pair was considered a random effect in the statistical analysis (“cross genotype”, see below).

Reproductive time-window and general husbandry conditions of these crosses were the same as for the experimental evolution protocol.

### Establishment of isofemale lines and generation of flies for the analysis of mitochondrial contribution to different phenotypes

To analyze the contribution of mitochondria associated with different *w*Mel clades to the fitness-related phenotypes we established *Wolbachia*-free lines derived from the above selected isofemale lines carrying different *w*Mel variants.

We treated ten clade III isofemale lines and ten clade V isofemale lines with tetracycline (as in [14]). Lines were raised in fly food with 0.05 mg/ml of tetracycline hydrochloride (Sigma) for two generations. After antibiotic treatment each treated line had their microbiota reconstituted with the microbiota associated with their original line. 150 μl of a bacterial inoculum of each of the original lines was added to each tetracycline-treated lines. Each inoculum was constituted of 5ml of sterile water mixed with 2 g of food from a 10 days old vial of the original stock, filtered to remove eggs and larvae.

All stocks were confirmed to be free of *Wolbachia* by PCR using primers specific for the *Wolbachia* gene *wsp*; wsp-81F (5’-TGGTCCAATAAGTGATGAAGAAAC-3’) and wsp-691R (5’-AAAAATTAAACGCTACTCCA-3’), as in [3]. Flies were raised without antibiotics for two generations before assays.

To compare the phenotype of different cytotypes in the presence or absence of *Wolbachia* we set up reciprocal crosses between lines carrying different *w*Mel variants and reciprocal crosses between their matching isofemales lines after tetracycline treatment. Only ten reciprocal crosses of each kind were performed in this assay. The phenotypic assays were performed on the progeny of these crosses.

### Fitness assays

For the survival assays, 100 females (3–6 days old) from each reciprocal cross, infected with DCV, were placed in vials (10 vials with 10 individuals each), at 25°C. The mortality was monitored daily for 20 days.

For the progeny assays, 20 couples (3–6 days old) from each reciprocal cross were infected with DCV and placed in vials 5 days after infection (1 couple per vial). Flies were allowed to lay eggs for two days and then removed (this protocol matches that of the experimental evolution). The progeny of each female corresponds to the number of pupae per vial. The same protocol was used for progeny quantification with females not exposed to DCV.

### *Wolbachia* levels and viral titers

For the quantification of *Wolbachia* and viral titers in the progeny of the reciprocal crosses, we used three DCV-infected females of the progeny of each matched pair. Seven days post-infection total nucleic acid was extracted using MasterPure Complete DNA and RNA Purification Kit (Epicentre), according to manufacturers' protocol, with some modifications. To purify DNA, 10 μl of each sample was treated with 1 μl of 10 mg/ml RNAse A (Roche). To purify RNA, samples were treated with 1U DNAse (Promega) per μg of total nucleic acid, in a total volume of 10 μl, at 37°C for 30 min; the reaction was stopped by adding 1 μl of RQ1 DNAse stop solution, and incubated at 65°C to inactivate the DNAse. RNA samples were then reverse transcribed to cDNA using M-MLV Reverse Transcriptase (Promega), according to manufacturers’ instructions. DNA and cDNA samples were used to quantify *Wolbachia* and DCV levels, respectively.

Quantification of *Wolbachia* levels and viral titers was performed by qPCR as described in [14]. For each reaction we used 6 μl of iQTM SYBR Green supermix (Bio-Rad), 0.5 μl of each primer solution at 3.6 μM and 5 μl of diluted DNA. Each plate contained three technical replicates of every sample for each set of primers. Relative amounts of *wsp* and DCV were calculated using the Pfaffl method [53] and *Drosophila Rpl32* as a reference. Levels of *wsp* and DCV are relative to the *w*Mel clade V samples.

### Statistical analysis

#### Allele frequency comparisons

Allele frequencies were compared using a weighted binomial model. Let *ν*_*i*_ be the frequency of the major allele in population *i* of a given selection regime:

$$v_{i}={{logit}^{-1}\;(X}_{i}^{T}\beta +\varepsilon_{i})$$ (1)

Where β is the vector of the Selection regime fixed effect and *X*_*i*_ is a row vector relating this fixed effect to population *i*, weighted by the read depth or number of genotyped individuals. *ε*_*i*_ is the residual error that captures overdispersion in the estimate of frequencies in each population.

In the pooled sequencing analysis, only positions in any of the populations with minor allele frequency > 2% were considered, and Benjamini & Hochberg adjusted *p* values (q-values) were considered significant if below a false discovery rate threshold of 0.1%.

In the comparisons of *w*Mel variant frequencies between selection regimes, *p* values for multiple comparisons were adjusted using sequential Bonferroni correction.

In generation 20 all reads or all sampled individuals in the Virus-Selected populations were fixed to one of the alleles, leading to problems of convergence in the models. To correct for that, we assigned one read or one individual to the alternative allele.

#### Estimation of selection coefficient

Since *Wolbachia* is maternally transmitted, selection acts as in a haploid organism. The estimate for the fitness differential between the *Wolbachia* clades in the Virus-Selected populations was therefore calculated according to [31] (eqn 6.3):

$$\log\;\left(\frac{p_{t}}{q_{t}}\right)=\log\;\left(\frac{p_{0}}{q_{0}}\right)+\log\left(w\right)*t$$ (2)

Where *w* is the relative fitness (1-*s*) of genotype *p* over genotype *q*.

Assuming a small *s* (<0.5), (1+*s*)^*t*^ ~ *e*^*st*^. Therefore, we assessed statistical significance of the coefficient using mixed logistic regression. Let *ν*_*i*,*t*_ be the frequency of a given *Wolbachia* genotype at generation *t* in population *i*,

$$v_{i,t}={logit}^{-1}(s*t+\nu_{i,0}+\varepsilon_{i,t})$$ (3)

The selection coefficient (*s*) is the slope of the regression coefficient given the initial frequencies (*ν*_*i*,*0*_), *ε*_*i*,*t*_ is the residual error that captures overdispersion in the estimate of frequencies in the populations at each time point. Using the frequencies of *w*Mel variants at generations 0, 5, 10 and 20 the selection coefficient against *w*Mel I/III is 0.263 (0.177–0.349). This relatively high value is independent of the data at generation 20, when there is fixation of clade V, since the selection coefficient calculated with data from generations 0, 5, and 10 is 0.287 (0.183–0.391).

We tested for the presence of *w*Mel in the progeny (96 individuals) of five females from different isofemale lines carrying clade III variants, and in the progeny (100 individuals) of five females from different isofemale lines carrying clade V variants. All individuals were positive for *w*Mel showing that vertical transmission is virtually 100% and similar for variants of both clades. Therefore, we can compare these variants fitness using this multigenerational equation.

#### Survival analysis

To compare survival at days 5, 6 and 7 of flies with each *Wolbachia* variant after infection, we fitted a generalized linear mixed effects model. Let *ν*_*i*,*j*_ be the proportion of surviving flies in vial *i* of individuals of a given *w*Mel variant, resulting from cross *j*, at 5, 6 or 7 d.p.i.:

$$v_{i,j}={{logit}^{-1}\;(X}_{i}^{T}\beta +c_{j}+\varepsilon_{i,j})$$ (4)

Where *β* is the vector of fixed effects of *w*Mel variant, *X*_*i*_ is the row vector relating the fixed effects of variant with vial, *c*_*j*_ is the random effect of fly cross genotype and *ε*_*i*,*j*_ is the residual error that captures overdispersion for each vial.

We also compared the full survival dynamics, until 20 days post-infection, using a mixed effects Cox model. This model accounted for both parental cross and between-vial variation in survival rates. The hazard of the *i*th individual of a given *w*Mel strain, resulting from cross *j* in vial *k* was modeled as:

$$H_{i,j,k}(t)=H_{0}(t)e^{X_{i}^{T}\beta +c_{j}+\varepsilon_{i,j,k}}$$ (5)

Where *H*_*0*_ is the baseline hazard at time *t*, β is the vector of fixed effects of *w*Mel variant, *X*_*i*_ is the row vector relating the fixed effects of variant with the individual fly, *c*_*j*_ is the random effect of cross genotype and *ε*_*i*,*j*,*k*_ is the random effect of vial.

In both analyses, the effect of the *w*Mel variant was compared using likelihood-ratio tests, with a model without the fixed effect term as the null model.

To analyze the effect of the mitochondria variants in the survival upon viral infection equivalent models were used taking into account the fixed effect of presence or absence of *Wolbachia*, and its interaction with the fixed effect variant.

#### Reproduction tests

To compare reproductive output of flies with different *w*Mel variants after infection, we fitted a linear mixed model, where *ν*_*i*,*j*_ is the number of pupae after a 48h oviposition period by female *i* resulting from cross *j*, with a particular *w*Mel variant.

$$v_{i,j}=X_{i}^{T}\beta +c_{j}+\varepsilon_{i,j}$$ (6)

As above, β is the vector of fixed effects of *w*Mel variant, *X*_*i*_ is the row vector relating the fixed effects of *w*Mel variant with female *i*, *c*_*j*_ is a random variable representing the deviation of the cross genotype (reciprocal cross pair) from the overall mean and *ε*_*i*,*j*_ is the random term that captures heterogeneity between different females of the same cross genotype.

The effect of the fixed factor was compared using likelihood-ratio tests.

To analyze the effect of the mitochondria variants in the reproductive output upon viral infection, a similar model was used taking into account the fixed effect of presence or absence of *Wolbachia*, and the interaction of this with the fixed effect variant.

In the second experiment (designed to test for the effect of mitochondria) there was a high number of females that did not reproduce. Therefore, we also analyzed these data using a hurdle model for count data in which two equations were used; one to compare the number of zero vs non-zero counts between the groups with a binomial model, and another to analyze the non-zero counts, assuming that these follow a zero-truncated negative binomial distribution. This analysis gave a similar result to the linear mixed model used above. In the non-zero counts data there was an interaction between cytotype and *Wolbachia* presence (χ^2^_1_ = 9.59, *p* = 0.002). There was a significant difference in reproductive output between flies carrying *w*Mel clade V or *w*Mel clade III, but not between flies of the same cytotypes without *Wolbachia* (pairwise comparisons between clades in generalized linear mixed model, *t* = 5.23, *p* < 0.001 and *t* = 1.71, *p* = 0.087, for flies with and without *Wolbachia*, respectively).

#### Wolbachia and DCV titer quantification

To compare *Wolbachia* or DCV titers after infection in flies with different *w*Mel variants, we fitted a linear mixed model similar to the Eq (6), with *ν*_*i*,*j*_ being the log(*wsp*) or log(DCV) levels.

All statistical analyses were done in R version 3.1.2 [54]. Linear mixed models were fitted using the *lmer* function and generalized linear mixed models with the *glmer* function, both in the “lme4” package. Hurdle models were done with the *glmmADMB* function of the “glmmADMB” package. Multiple comparisons were done using the *lsmeans* function in the “lsmeans” package. Survival data were compared using the *coxme* function in “coxme” package.

### Accession numbers

Trimmed fastq and assembled bam files are available via the European Nucleotide Archive (http://www.ebi.ac.uk/ena/about/search_and_browse), as project PRJEB8815, with reads accession numbers ERS684186-ERS684197 and ERS764859–ERS764870, respectively.

## Supporting Information

S1 Text Supplementary analysis of Pool-Seq data and frequencies of *w*Mel variants. (DOCX)

S1 Fig Frequencies of significantly differentiated SNPs in the Ancestral, Control and Virus-Selected populations. Frequencies of the major allele of *Wolbachia* single nucleotide polymorphisms (SNPs) in Ancestral (top), Control (middle) and Virus-Selected populations (bottom), determined by Pool-Seq. Shown are SNPs which significantly changed frequencies between Ancestral and Virus-Selected populations at generation 20. Panel columns 1 to 4 represent replicate populations.
(TIF)

S2 Fig Significantly different SNP frequencies between Ancestral, Control and Virus-Selected populations. **(A)** -log10 Benjamini & Hochberg adjusted *p* values for differences in frequencies of SNPs across the *w*Mel genome, between the Ancestral and Control populations (top panel), Ancestral and Virus-Selected populations (mid panel) and Control and Virus-Selected populations (bottom panel). All the SNPs that were polymorphic in the Pool-Seq analysis are shown. **(B)** Venn diagram of the overlap of the significantly differentiated SNPs between Ancestral, Control and Virus-Selected populations. SNP frequencies were considered significantly different among treatments when Benjamini & Hochberg adjusted *p* values (q-values) were below a false discovery rate threshold of 0.1%.
(TIF)

S3 Fig *Wolbachia* variants, not mitochondrial variants, confer differential protection to viral infection. **(A)** Survival of flies of clade V (dark gray) and clade III (light gray) cytotypes five, six and seven days post infection with DCV (d.p.i.), in the presence or absence of *w*Mel. **(B)** Cox hazard ratio between flies of clade V and clade III cytotypes, calculated from survival data until 20 d.p.i., in the presence (Wolb+) or absence of *w*Mel (Wolb-). **(C)** Reproductive output of 5–7 d.p.i. flies of clade V and clade III cytotypes, in the presence (Wolb+) or absence of *w*Mel (Wolb-). In all assays the female progeny of ten independent reciprocal crosses between isofemale flies of clade V (dark gray) and clade III (light gray) genotypes, were analyzed after systemic infection with DCV (2 x 10^7^ TCID_50_/ml). ***-*p* < 0.001. Means (± 95% confidence intervals) are shown in all panels.
(TIF)

S4 Fig *Drosophila* reproductive output is not influenced by the *Wolbachia* variant in the absence of DCV infection. Mean (±95% confidence interval) reproductive output of flies carrying Clade V and Clade III *w*Mel variants in the absence of DCV infection. Reproductive output between flies carrying different *w*Mel variants is not significantly different (GLMM, χ2_1_ = 2.322, *p* = 0.128). The female progeny of eleven independent reciprocal crosses between isofemale flies, carrying Clade V and Clade III *w*Mel variants, were assayed. Females 8–11 day-old laid eggs for 48h, matching the protocol of DCV infected flies.
(TIF)

S5 Fig DCV and *Wolbachia* loads in flies carrying *w*Mel variants from different clades. **(A)** Relative DCV levels at 7 d.p.i. and **(B)** relative *Wolbachia* titers 7 d.p.i. The female progeny of eleven reciprocal crosses between isofemale flies, carrying Clade V and Clade III *w*Mel variants, were analyzed after systemic infection with DCV (2 x 10^7^ TCID_50_/ml). *p* < 0.001 (***) in both comparisons.
(TIF)

S6 Fig Frequencies of *w*Mel Clade V diagnostic SNPs in the Ancestral, Control and Virus-Selected populations. Frequencies of *w*Mel Clade V diagnostic SNPs in Ancestral, Control and Virus-Selected populations, determined by Pool-Seq. The data is discriminated for the four replicate populations of each condition.
(TIF)

S1 Dataset Polymorphic positions in the *w*Mel genome in Ancestral, Control and Virus-Selected populations. Frequencies of the 211 positions that were detected as being polymorphic (major allele frequency ≤ 0.98) in any of the pooled samples. Shown are position in the reference *w*Mel genome (AE017196) (**POS**), nucleotide in reference genome (**REF**), alternative nucleotide (**ALT**), major allele in Ancestral populations (**MA_C0**) and frequencies in the Ancestral (**C0**), Control (**C20**) or Virus-Selected (**V20**) columns (95% confidence intervals are shown in the corresponding _**CI** columns). The frequencies of these SNPs in each population are discriminated in the columns **C0_1–4**, **C20_1–4**, and **V20_1–4**. 133 of these polymorphisms significantly differentiated in at least one of the 3 possible comparisons between Ancestral, Control and Virus-Selected regimes (“Sign” in the **C0_C20**, **C0_V20** and **C20_V20** columns). Benjamini & Hochberg adjusted *p* values (q-values) were considered significant if below a false discovery rate threshold of 0.1%. Columns **Clades I-VIII** show the allele(s) described for these clades in [13,14,21]. In all positions, the major allele in the Ancestral populations (MA_C0) matched the one described for clade V [21]. The column **Annotation** indicates in which predicted gene the SNP is located or if it is in an intergenic region. For each SNP within a gene the change in the codon (**Codon**) and if that leads to a synonymous or non-synonymous codon (**Effect**) is shown.
(CSV)

S2 Dataset Mean survival across generations in the Virus and Bacteria Selection regimes. Estimates for the mortality after infection of individuals from Virus- and Bacteria-Selected populations, across 20 Generations of selection. Average of log-odds across all populations and both sexes (**Mean**), and separately for each of the four replicate populations (**Pop_1–4**) are shown. Female and male survival estimates are shown in the **F_Pop_1–4** and **M_Pop_1–4** columns, respectively. Number of total (**N_Total**), female (**N_Females**) and male (**N_males**) selected individuals, are shown. (NA) represent estimates of survival, which are not available (e.g. females at generations 0–3), despite having been selected. (NS) represent generations where selection was not done.
(CSV)

S3 Dataset Frequencies of flies carrying *w*Mel clade III or clade V in Ancestral populations and generation 20 of Control, Virus-Selected, and Bacteria-Selected populations (Data for Fig 1B). (TXT)

S4 Dataset Frequencies of flies carrying *w*Mel clade III or clade V in Ancestral populations, and Virus-Selected populations at several generations (Data for Fig 1C). (TXT)

S5 Dataset Survival data for DCV-infected flies carrying different *w*Mel clades (Data for Fig 2A and 2B). (TXT)

S6 Dataset Reproduction data for DCV-infected flies carrying different *w*Mel clades (Data for Fig 2C). (TXT)

S7 Dataset Survival data for DCV-infected flies with different cytotypes, with and without *w*Mel (Data for S3A and S3B Fig). (TXT)

S8 Dataset Reproduction data for DCV-infected flies with different cytotypes, with and without *w*Mel (Data for S3C Fig). (TXT)

S9 Dataset Reproduction data for non-infected flies carrying different *w*Mel clades (Data for S4 Fig). (TXT)

S10 Dataset Relative DCV titers in flies carrying different *w*Mel clades (Data for S5A Fig). (TXT)

S11 Dataset Relative *Wolbachia* titers in flies carrying different *w*Mel clades (Data for S5B Fig). (TXT)
