# Supplementary material for: Drosophila Adaptation to Viral Infection through Defensive Symbiont Evolution
Source: PLoS Genet. 2016 Sep 29;12(9):e1006297. doi: 10.1371/journal.pgen.1006297 (PMC5042464; doi:10.1371/journal.pgen.1006297)
Supplement: S1 Text — (DOCX) [file pgen.1006297.s001.docx]

**S1 Text. Supplementary analysis of Pool-Seq data and frequencies of *w*Mel variants.**

***Wolbachia* coverage analysis.** Genome coverage of *Wolbachia* in the resequenced populations was almost complete, with 96% of the positions being covered at least by one read. 93% of all positions passed the defined coverage quality cutoff (±2SD of the mean coverage of the positions in the *Wolbachia* genome with 1 or more mapped reads) in all populations. Average *Wolbachia* genome coverage was between 124 and 286. Fluctuations in coverage matched those of the *Drosophila* genome in the same samples. From the 600 non-ambiguous polymorphic positions described previously [13,14,21] only 11 did not pass the coverage quality cutoff. Our data therefore contain information on >93% of the *Wolbachia* genome and >98% of the previously described SNPs.

**Determining *Wolbachia* haplotypes from Pool-Seq data.** For this analysis we focused on the polymorphic nucleotide positions (i.e., in which the major allele frequency was ≤0.98) in any of the three conditions (Fig 1, S1 Fig, S2 Fig, S1 Dataset). In the Ancestral populations we identified single nucleotide polymorphisms (SNPs) that only occur in *Wolbachia* variants of three of the major clades of wMel [13,14,21]. Fifteen of the polymorphic positions are SNPs of clade I variants. One of these SNPs is diagnostic of this clade (i.e. it is present in all variants of this clade alone) and had a median frequency of 0.038. Twelve SNPs have only been detected in clade III variants, three of which are diagnostic and had a median frequency of 0.082 (0.072-0.097). Forty-seven SNPs are diagnostic of clade V and had a median frequency of 0.891 (0.860-0.919). We have not found SNPs exclusive of any other major clade (S1 Dataset). The sum of the medians of these frequencies is very close to one (1.011), indicating that the ancestral *Wolbachia* population was comprised of approximately 88% clade V variants and 12% of variants of clades I and III.

**Fixation of clade V variants is a specific response to DCV challenge.** Control populations that evolved under mock infection did not show the same pattern of change in clade V variants as Virus-Selected populations. Clade V specific SNPs had a median frequency of 0.891 (0.860-0.919) in the Ancestral populations, and of 0.872 (0.837-0.903) in the Control populations. The frequency of each of these SNPs did not change significantly between Ancestral and Control populations.

In fact, only 8 SNPs significantly changed between Ancestral and Control populations, which have only been detected before in clades I and III (S1 Dataset). The frequency of the SNP diagnostic of clade I *w*Mel variants in the Ancestral populations had a median of 0.038, while in the Control populations the median was 0.007. SNPs diagnostic of clade III had a median frequency of 0.082 (0.072-0.097) in the Ancestral populations and 0.125 (0.123-0.131) in the Control populations. Therefore, *w*Mel variants from clade I seem to have been eliminated from Control populations and replaced by those of clade III. These results indicate a difference in the frequencies of clade I and clade III variants between Ancestral and Control populations, but not in frequencies of clade V *w*Mel variants.

**Dynamics of clade V *w*Mel variants.** From the PoolSeq data the averages of the 47 diagnostic SNPs of clade V *w*Mel variants vary between 0.784 and 0.930 in the different replicates of Control and Ancestral populations (S6 Fig). However, the standard deviation of frequency of these 47 SNPs is low in all the populations (between 0.017 and 0.033). In all the Virus-selected populations the average of these SNPS frequency is 0.999 and the standard deviation is 0.002 or below. The parallel behavior of all these diagnostic SNPs, which are spread throughout the *w*Mel genome, clearly supports the idea that they are completely linked and that selection is acting on *w*Mel haplotypes without recombination.

We also followed, by PCR restriction analysis, the frequency of a SNP in position 805,011, which differs between clades I, II, III, IV and VIII, on one hand, and clades V and VI, on the other. Thus, we can use it in our populations to distinguish flies with *w*Mel variants of clade V from flies with variants of clade I/III. Based on this assay, the frequency of clade V variants in the Ancestral populations (Generation 0) was 0.842 (0.802-0.875) and in the Control populations was 0.815 (0.728-0.879). These estimates are in agreement with the Pool-Seq estimate of 0.891 (0.86-0.92) and 0.872(0.84-0.90), respectively. At generation 20 clade V was fixed in Virus-Selected populations, also confirming the Pool-Seq data.

**Absence of within clade V selection.** In the Virus-Selected populations there were still 57 polymorphic positions, with the major allele frequencies ranging from 0.55 to 0.97 (S1 Dataset). Fifty-six of these SNPs are new and one has been described before in clades V and VI. This indicates that some polymorphisms occur within clade V and that different haplotypes exist in these populations. Of these 57 SNPs, two show a small but significant change between Ancestral and Virus-Selected populations. In position 611,262 the major allele changes from 0.917 to 0.834, and in position 739,559 the major allele frequency changes from 0.624 to 0.747. The frequency of the other 55 SNPs did not change significantly. These results indicate that there is no strong selection between *w*Mel variants within clade V, upon viral challenge.

**Description of the SNPs fixed between Ancestral and Virus-selected populations.** The 123 SNPs that became fixed between Ancestral and Virus-selected populations are spread throughout the genome of *w*Mel (from positions 10,452 to 1,257,121 of the 1,267,782 nucleotides genome [46]). Thirty-four of these SNPs are in intergenic regions and 89 in predicted coding regions, 67 of these are non-synonymous substitutions (S1 Dataset).
