# Supplementary figures and images for: Drosophila Adaptation to Viral Infection through Defensive Symbiont Evolution
Source: PLoS Genet. 2016 Sep 29;12(9):e1006297. doi: 10.1371/journal.pgen.1006297 (PMC5042464; doi:10.1371/journal.pgen.1006297)

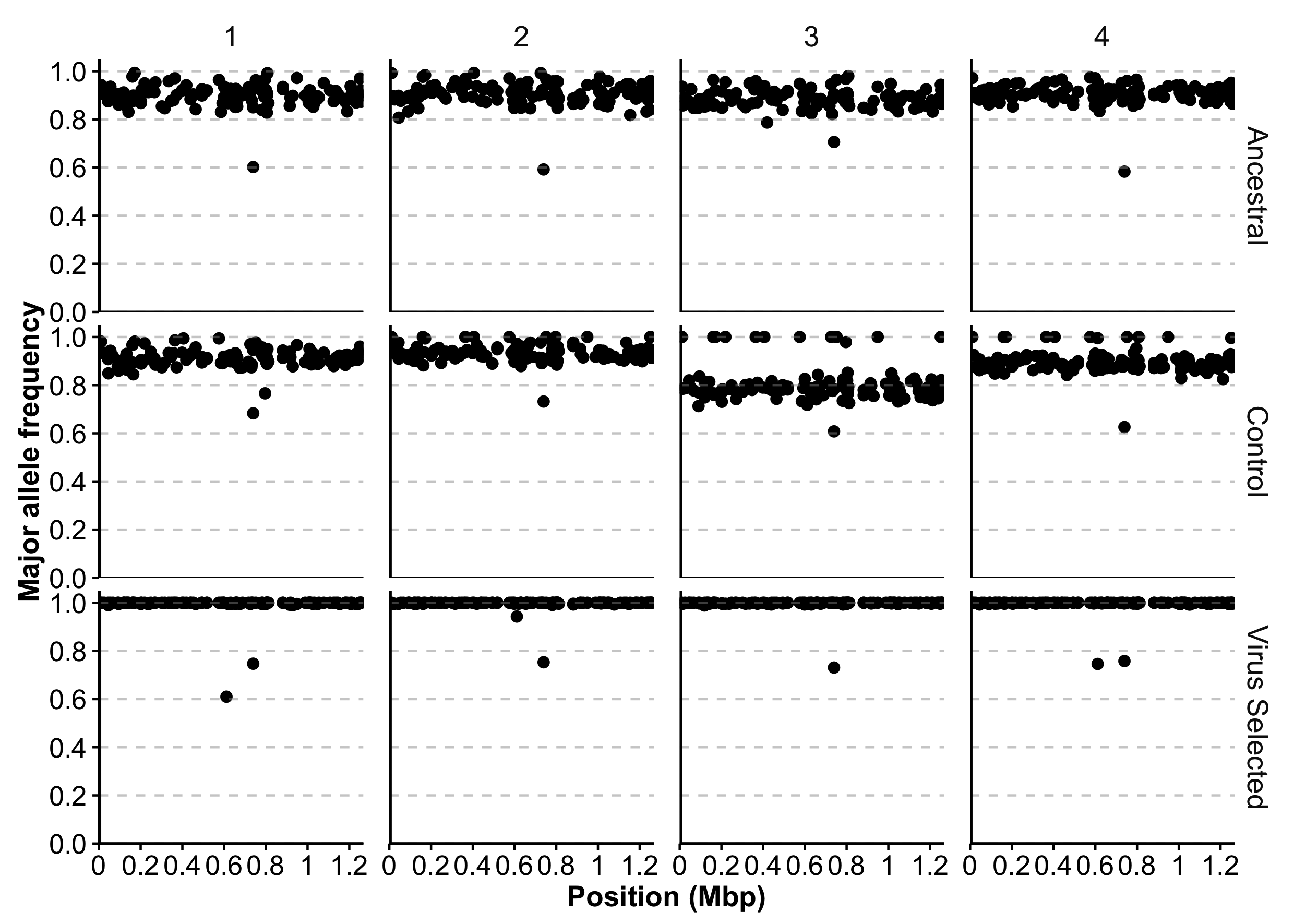

Supplement: S1 Fig — Frequencies of the major allele of Wolbachia single nucleotide polymorphisms (SNPs) in Ancestral (top), Control (middle) and Virus-Selected populations (bottom), determined by Pool-Seq. Shown are SNPs which significantly changed frequencies between Ancestral and Virus-Selected populations at generation 20. Panel columns 1 to 4 represent replicate populations. (TIF) [file pgen.1006297.s002.tif]

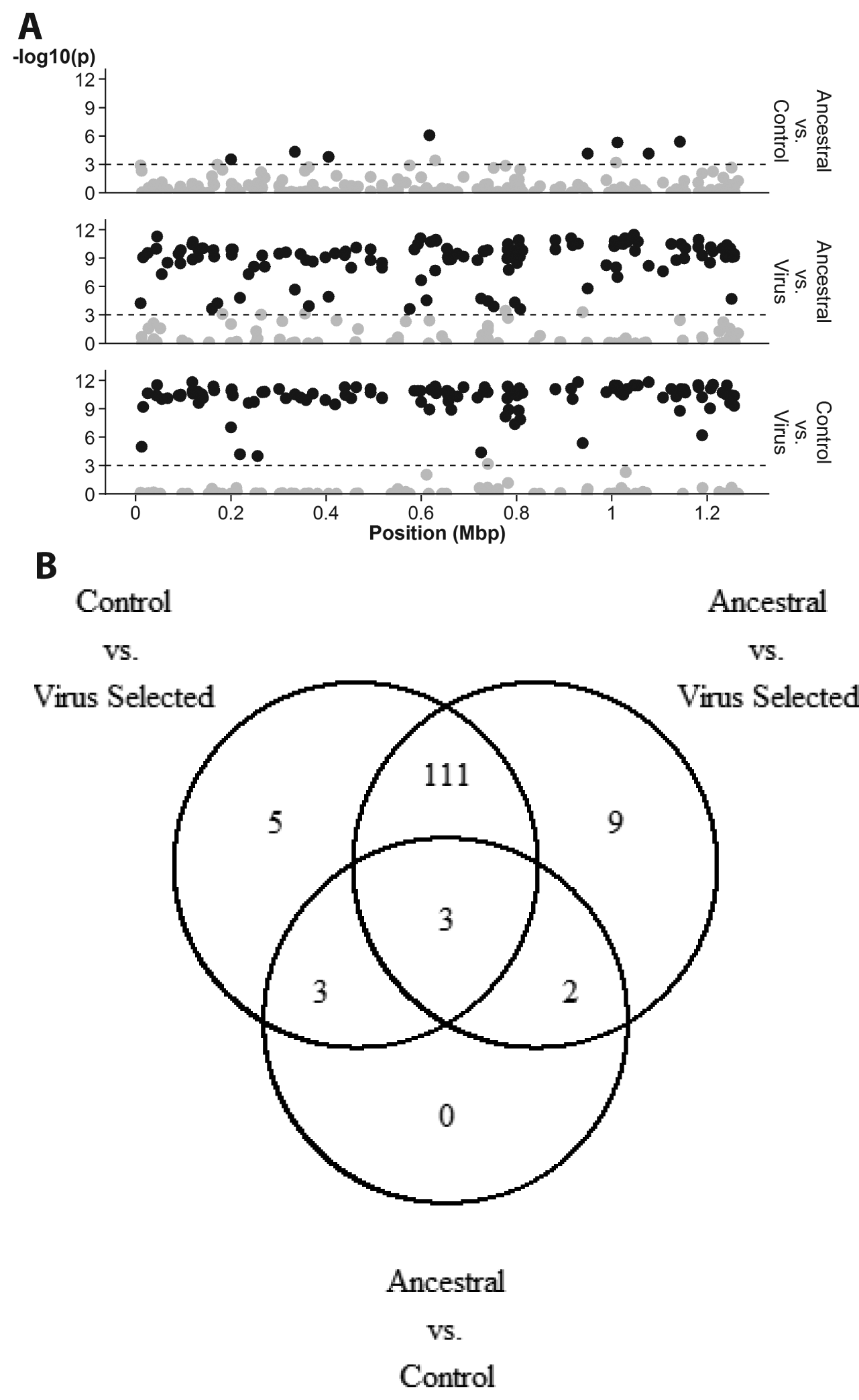

Supplement: S2 Fig — (A) -log10 Benjamini & Hochberg adjusted p values for differences in frequencies of SNPs across the wMel genome, between the Ancestral and Control populations (top panel), Ancestral and Virus-Selected populations (mid panel) and Control and Virus-Selected populations (bottom panel). All the SNPs that were polymorphic in the Pool-Seq analysis are shown. (B) Venn diagram of the overlap of the significantly differentiated SNPs between Ancestral, Control and Virus-Selected populations. SNP frequencies were considered significantly different among treatments when Benjamini & Hochberg adjusted p values (q-values) were below a false discovery rate threshold of 0.1%. (TIF) [file pgen.1006297.s003.tif]

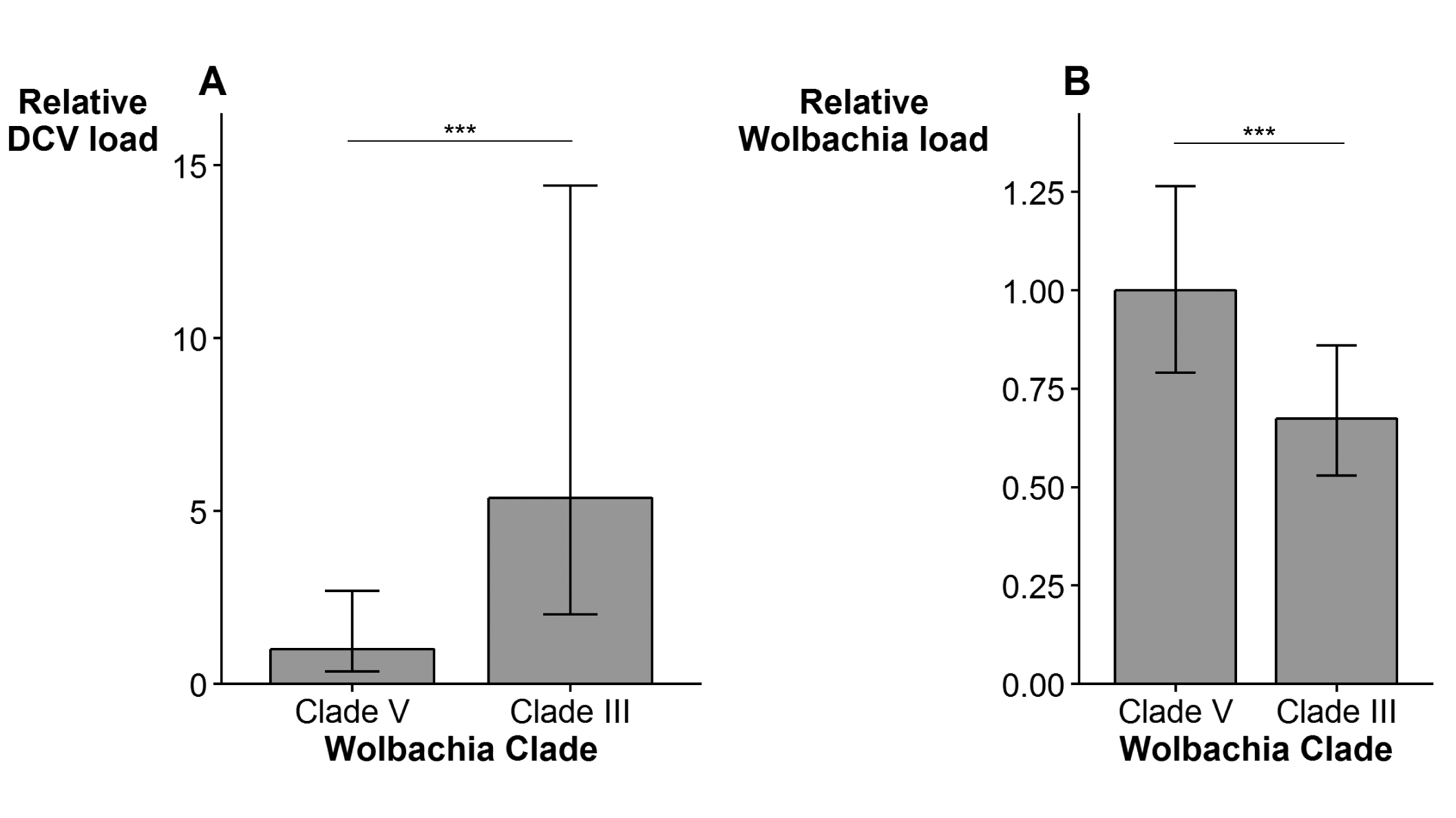

Supplement: S5 Fig — (A) Relative DCV levels at 7 d.p.i. and (B) relative Wolbachia titers 7 d.p.i. The female progeny of eleven reciprocal crosses between isofemale flies, carrying Clade V and Clade III wMel variants, were analyzed after systemic infection with DCV (2 x 10^7^ TCID50/ml). p < 0.001 (***) in both comparisons. (TIF) [file pgen.1006297.s006.tif]

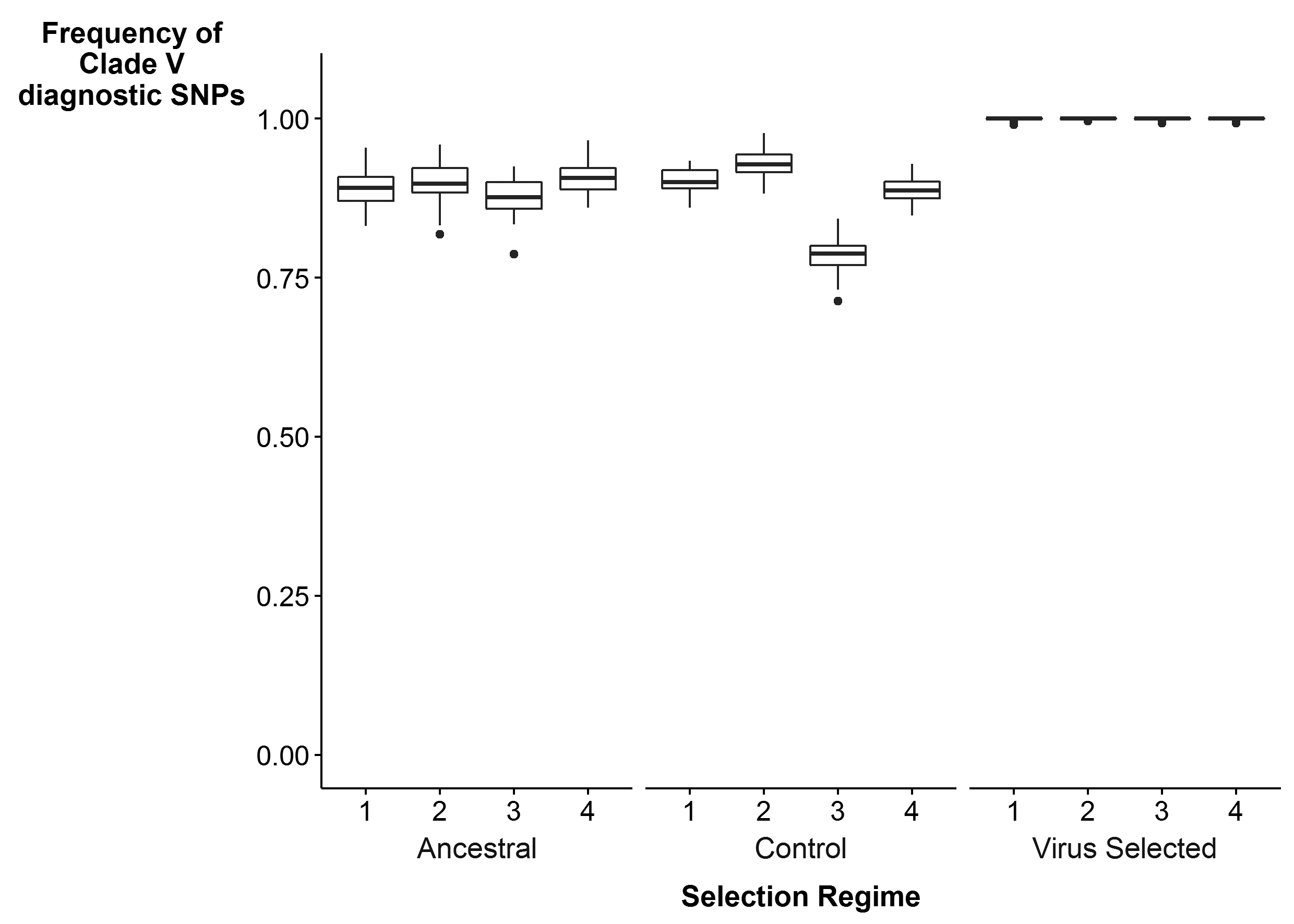

Supplement: S6 Fig — Frequencies of wMel Clade V diagnostic SNPs in Ancestral, Control and Virus-Selected populations, determined by Pool-Seq. The data is discriminated for the four replicate populations of each condition. (TIF) [file pgen.1006297.s007.tif]
